# 4-Bromo-3-methyl­anilinium perchlorate

**DOI:** 10.1107/S160053680903520X

**Published:** 2009-09-09

**Authors:** Li Zhang

**Affiliations:** a Ordered Matter Science Research Center, College of Chemistry and Chemical Engineering, Southeast University, Nanjing 210096, People’s Republic of China

## Abstract

In the title compound, C_7_H_9_BrN^+^·ClO_4_^−^, the cations and anions are linked by inter­molecular N—H⋯O hydrogen bonds, forming a two-dimensional network parallel to the *ab* plane.

## Related literature

For the use of amine derivatives in the construction of metal-organic frameworks, see: Fu *et al.* (2007 ▶, 2008 ▶); Fu & Xiong (2008 ▶); Wang *et al.* (2002 ▶). For applications of metal-organic coordination compounds, see: Chen *et al.* (2001 ▶); Xiong *et al.* (1999 ▶); Xie *et al.* (2003 ▶); Zhao *et al.* (2004 ▶). 
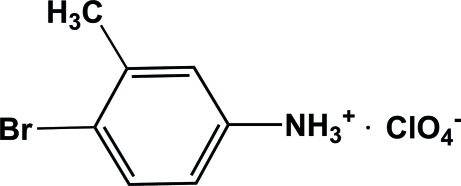


## Experimental

### 

#### Crystal data

C_7_H_9_BrN^+^·ClO_4_^−^
*M* *_r_* = 286.51
Triclinic, 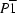

*a* = 4.9455 (10) Å
*b* = 6.9647 (14) Å
*c* = 15.714 (3) Å
α = 95.78 (3)°
β = 94.40 (3)°
γ = 102.62 (3)°
*V* = 522.8 (2) Å^3^
*Z* = 2
Mo *K*α radiation
μ = 4.18 mm^−1^
*T* = 298 K
0.40 × 0.05 × 0.05 mm

#### Data collection

Rigaku Mercury2 diffractometer
Absorption correction: multi-scan (*CrystalClear*; Rigaku, 2005 ▶) *T*_min_ = 0.910, *T*_max_ = 1.000
5411 measured reflections
2382 independent reflections
1584 reflections with *I* > 2σ(*I*)
*R*_int_ = 0.053

#### Refinement

*R*[*F* ^2^ > 2σ(*F* ^2^)] = 0.051
*wR*(*F* ^2^) = 0.127
*S* = 1.03
2382 reflections
129 parameters
H-atom parameters constrained
Δρ_max_ = 0.44 e Å^−3^
Δρ_min_ = −0.57 e Å^−3^

### 

Data collection: *CrystalClear* (Rigaku, 2005 ▶); cell refinement: *CrystalClear*; data reduction: *CrystalClear*; program(s) used to solve structure: *SHELXS97* (Sheldrick, 2008 ▶); program(s) used to refine structure: *SHELXL97* (Sheldrick, 2008 ▶); molecular graphics: *SHELXTL* (Sheldrick, 2008 ▶); software used to prepare material for publication: *SHELXTL*.

## Supplementary Material

Crystal structure: contains datablocks I, global. DOI: 10.1107/S160053680903520X/ci2895sup1.cif

Structure factors: contains datablocks I. DOI: 10.1107/S160053680903520X/ci2895Isup2.hkl

Additional supplementary materials:  crystallographic information; 3D view; checkCIF report

## Figures and Tables

**Table 1:** Hydrogen-bond geometry (Å, °)

*D*—H⋯*A*	*D*—H	H⋯*A*	*D*⋯*A*	*D*—H⋯*A*
N1—H1*A*⋯O2^i^	0.89	2.04	2.909 (4)	166
N1—H1*B*⋯O1^ii^	0.89	2.03	2.875 (4)	159
N1—H1*C*⋯O3	0.89	2.02	2.857 (4)	156

Symmetry codes: (i) 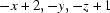; (ii) 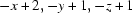.

**Fig. 1.**
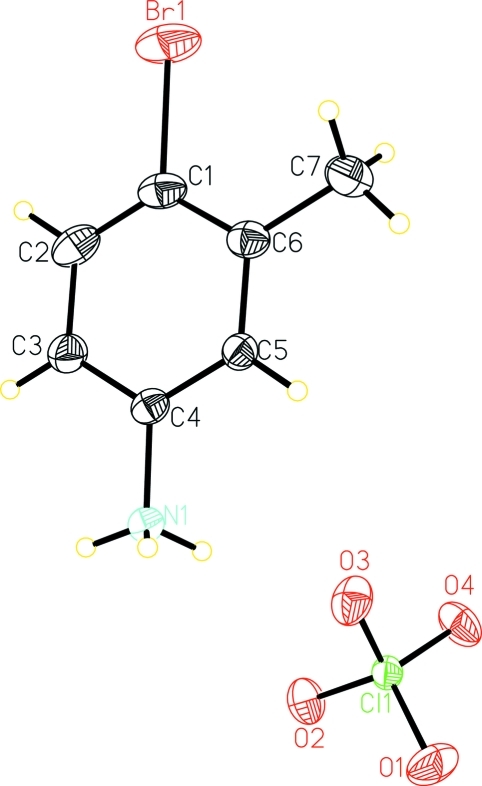
The molecular structure of the title compound, with the atomic numbering scheme. Displacement ellipsoids are drawn at the 30% probability level.

**Fig. 2.**
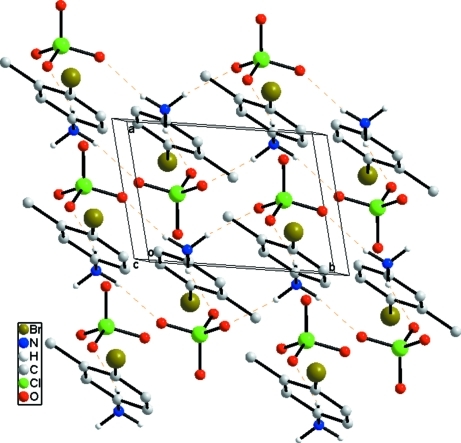
The crystal packing of the title compound, viewed along the c axis, showing the N—H···O hydrogen bonds (dashed lines). H atoms not involved in hydrogen bonding have been omitted for clarity.

## supplementary crystallographic information

### Comment

The construction of metal-organic coordination compounds has attracted much attention owing to potential functions, such as permittivity, fluorescence, magnetism and optical properties (Chen *et al.*, 2001; Xie *et al.*, 2003; Zhao *et al.*, 2004; Xiong *et al.*, 1999). Amine derivatives are a class of excellent ligands for the construction of novel metal-organic frameworks (Fu *et al.*, 2007, 2008; Wang *et al.* 2002; Fu & Xiong 2008). We report here the crystal structure of the title compound, 4-bromo-3-methylanilinium perchlorate.

In the title compound (Fig.1), the amino N atom is protonated. In the crystal structure, all the amine group H atoms are involved in N—H···O hydrogen bonding (Table 1) with O atoms of ClO_4_^-^ anion. These hydrogen bonds link the ionic units into a two-dimensional network parallel to the *ab* plane (Fig. 2).

### Experimental

The commercial 4-bromo-3-methylbenzenamine (3 mmol, 0.75 g) and HClO_4_ (0.5 ml) were dissolved in ethanol (20 ml). Colourless block-shaped crystals of the title compound suitable for X-ray analysis were obtained by slow evaporation at room temperature.

### Refinement

H atoms were positioned geometrically and treated as riding [C-H = 0.93 Å (aromatic), 0.96 Å (methyl) and N-H = 0.89 Å (N)], with *U*_iso_(H) = 1.2*U*_eq _(aromatic C) and 1.5*U*_eq_(methyl C or N). A rotating-group model was used for the methyl and -NH_3_ groups.

### Crystal data

**Table:**

C_7_H_9_BrN^+^·ClO_4_^−^	*Z* = 2
*M**_r_* = 286.51	*F*(000) = 284
Triclinic, *P*1	*D*_x_ = 1.820 Mg m^−^^3^
Hall symbol: -P 1	Mo *K*α radiation, λ = 0.71073 Å
*a* = 4.9455 (10) Å	Cell parameters from 1584 reflections
*b* = 6.9647 (14) Å	θ = 3.0–27.5°
*c* = 15.714 (3) Å	µ = 4.18 mm^−^^1^
α = 95.78 (3)°	*T* = 298 K
β = 94.40 (3)°	Needle, colourless
γ = 102.62 (3)°	0.40 × 0.05 × 0.05 mm
*V* = 522.8 (2) Å^3^	

### Data collection

**Table:**

Rigaku Mercury2 diffractometer	2382 independent reflections
Radiation source: fine-focus sealed tube	1584 reflections with *I* > 2σ(*I*)
graphite	*R*_int_ = 0.053
Detector resolution: 13.6612 pixels mm^-1^	θ_max_ = 27.5°, θ_min_ = 3.0°
CCD profile fitting scans	*h* = −6→6
Absorption correction: multi-scan (*CrystalClear*; Rigaku, 2005)	*k* = −9→9
*T*_min_ = 0.910, *T*_max_ = 1.000	*l* = −20→20
5411 measured reflections	

### Refinement

**Table:**

Refinement on *F*^2^	Primary atom site location: structure-invariant direct methods
Least-squares matrix: full	Secondary atom site location: difference Fourier map
*R*[*F*^2^ > 2σ(*F*^2^)] = 0.051	Hydrogen site location: inferred from neighbouring sites
*wR*(*F*^2^) = 0.127	H-atom parameters constrained
*S* = 1.03	*w* = 1/[σ^2^(*F*_o_^2^) + (0.0535*P*)^2^ + 0.0338*P*] where *P* = (*F*_o_^2^ + 2*F*_c_^2^)/3
2382 reflections	(Δ/σ)_max_ = 0.001
129 parameters	Δρ_max_ = 0.44 e Å^−^^3^
0 restraints	Δρ_min_ = −0.56 e Å^−^^3^

### Special details

**Table:**

Geometry. All esds (except the esd in the dihedral angle between two l.s. planes) are estimated using the full covariance matrix. The cell esds are taken into account individually in the estimation of esds in distances, angles and torsion angles; correlations between esds in cell parameters are only used when they are defined by crystal symmetry. An approximate (isotropic) treatment of cell esds is used for estimating esds involving l.s. planes.
Refinement. Refinement of F^2^ against ALL reflections. The weighted R-factor wR and goodness of fit S are based on F^2^, conventional R-factors R are based on F, with F set to zero for negative F^2^. The threshold expression of F^2^ > 2sigma(F^2^) is used only for calculating R-factors(gt) etc. and is not relevant to the choice of reflections for refinement. R-factors based on F^2^ are statistically about twice as large as those based on F, and R- factors based on ALL data will be even larger.

### Fractional atomic coordinates and isotropic or equivalent isotropic displacement parameters (Å^2^)

**Table:**

	*x*	*y*	*z*	*U*_iso_*/*U*_eq_	
Br1	0.71423 (12)	0.24442 (9)	0.97211 (3)	0.0819 (3)	
N1	1.0957 (6)	0.2471 (5)	0.6136 (2)	0.0392 (7)	
H1A	1.1784	0.1471	0.6028	0.059*	
H1B	1.2142	0.3609	0.6090	0.059*	
H1C	0.9465	0.2317	0.5759	0.059*	
C1	0.8397 (8)	0.2542 (6)	0.8609 (3)	0.0470 (10)	
C2	0.9780 (10)	0.1151 (6)	0.8326 (3)	0.0548 (12)	
H2	1.0127	0.0226	0.8682	0.066*	
C3	1.0671 (9)	0.1102 (6)	0.7516 (3)	0.0461 (10)	
H3	1.1623	0.0160	0.7319	0.055*	
C4	1.0104 (8)	0.2496 (5)	0.7008 (2)	0.0351 (8)	
C5	0.8762 (7)	0.3934 (5)	0.7301 (2)	0.0357 (8)	
H5	0.8456	0.4876	0.6948	0.043*	
C6	0.7862 (8)	0.3996 (6)	0.8114 (3)	0.0412 (9)	
C7	0.6377 (9)	0.5534 (7)	0.8433 (3)	0.0582 (12)	
H7A	0.6417	0.6485	0.8029	0.087*	
H7B	0.7280	0.6191	0.8980	0.087*	
H7C	0.4479	0.4913	0.8493	0.087*	
Cl1	0.49638 (17)	0.22738 (12)	0.42344 (6)	0.0330 (2)	
O1	0.5691 (7)	0.3547 (4)	0.3590 (2)	0.0653 (9)	
O2	0.5586 (6)	0.0410 (4)	0.3998 (2)	0.0556 (8)	
O3	0.6498 (6)	0.3195 (4)	0.50396 (19)	0.0547 (8)	
O4	0.2066 (5)	0.1974 (4)	0.4320 (2)	0.0575 (8)	

### Atomic displacement parameters (Å^2^)

**Table:**

	*U*^11^	*U*^22^	*U*^33^	*U*^12^	*U*^13^	*U*^23^
Br1	0.0920 (5)	0.1064 (5)	0.0435 (4)	0.0025 (4)	0.0248 (3)	0.0218 (3)
N1	0.0411 (17)	0.0467 (18)	0.0321 (18)	0.0158 (15)	0.0029 (14)	0.0044 (15)
C1	0.046 (2)	0.055 (2)	0.034 (2)	−0.002 (2)	0.0072 (18)	0.006 (2)
C2	0.068 (3)	0.051 (2)	0.044 (3)	0.006 (2)	0.003 (2)	0.023 (2)
C3	0.056 (3)	0.044 (2)	0.041 (3)	0.018 (2)	0.004 (2)	0.0060 (19)
C4	0.0342 (18)	0.0356 (19)	0.032 (2)	0.0021 (16)	−0.0007 (16)	0.0044 (16)
C5	0.0339 (19)	0.039 (2)	0.036 (2)	0.0094 (16)	0.0042 (16)	0.0087 (17)
C6	0.038 (2)	0.045 (2)	0.036 (2)	0.0029 (18)	0.0026 (18)	0.0000 (18)
C7	0.059 (3)	0.071 (3)	0.048 (3)	0.022 (2)	0.011 (2)	−0.002 (2)
Cl1	0.0322 (4)	0.0332 (4)	0.0357 (5)	0.0104 (4)	0.0041 (4)	0.0074 (4)
O1	0.087 (2)	0.0574 (18)	0.055 (2)	0.0112 (18)	0.0140 (18)	0.0289 (16)
O2	0.0624 (19)	0.0415 (15)	0.068 (2)	0.0236 (15)	0.0084 (16)	0.0015 (14)
O3	0.0547 (18)	0.0522 (17)	0.0515 (19)	0.0132 (14)	−0.0179 (14)	−0.0049 (14)
O4	0.0305 (14)	0.072 (2)	0.068 (2)	0.0109 (14)	0.0101 (14)	−0.0056 (17)

### Geometric parameters (Å, °)

**Table:**

Br1—C1	1.902 (4)	C4—C5	1.379 (5)
N1—C4	1.464 (5)	C5—C6	1.384 (5)
N1—H1A	0.89	C5—H5	0.93
N1—H1B	0.89	C6—C7	1.493 (6)
N1—H1C	0.89	C7—H7A	0.96
C1—C2	1.362 (6)	C7—H7B	0.96
C1—C6	1.394 (6)	C7—H7C	0.96
C2—C3	1.377 (6)	Cl1—O2	1.419 (3)
C2—H2	0.93	Cl1—O4	1.421 (3)
C3—C4	1.378 (5)	Cl1—O1	1.428 (3)
C3—H3	0.93	Cl1—O3	1.437 (3)
			
C4—N1—H1A	109.5	C4—C5—C6	120.9 (4)
C4—N1—H1B	109.5	C4—C5—H5	119.5
H1A—N1—H1B	109.5	C6—C5—H5	119.5
C4—N1—H1C	109.5	C5—C6—C1	116.4 (4)
H1A—N1—H1C	109.5	C5—C6—C7	121.3 (4)
H1B—N1—H1C	109.5	C1—C6—C7	122.3 (4)
C2—C1—C6	122.6 (4)	C6—C7—H7A	109.5
C2—C1—Br1	117.7 (3)	C6—C7—H7B	109.5
C6—C1—Br1	119.7 (3)	H7A—C7—H7B	109.5
C1—C2—C3	120.6 (4)	C6—C7—H7C	109.5
C1—C2—H2	119.7	H7A—C7—H7C	109.5
C3—C2—H2	119.7	H7B—C7—H7C	109.5
C2—C3—C4	117.8 (4)	O2—Cl1—O4	108.72 (18)
C2—C3—H3	121.1	O2—Cl1—O1	109.8 (2)
C4—C3—H3	121.1	O4—Cl1—O1	109.9 (2)
C3—C4—C5	121.6 (4)	O2—Cl1—O3	110.55 (17)
C3—C4—N1	119.6 (3)	O4—Cl1—O3	109.01 (19)
C5—C4—N1	118.7 (3)	O1—Cl1—O3	108.79 (18)
			
C6—C1—C2—C3	−1.4 (7)	C4—C5—C6—C1	0.2 (5)
Br1—C1—C2—C3	178.0 (3)	C4—C5—C6—C7	−179.3 (4)
C1—C2—C3—C4	−0.2 (6)	C2—C1—C6—C5	1.4 (6)
C2—C3—C4—C5	1.8 (6)	Br1—C1—C6—C5	−178.0 (3)
C2—C3—C4—N1	−178.6 (3)	C2—C1—C6—C7	−179.2 (4)
C3—C4—C5—C6	−1.8 (6)	Br1—C1—C6—C7	1.4 (5)
N1—C4—C5—C6	178.6 (3)		

### Hydrogen-bond geometry (Å, °)

**Table:**

*D*—H···*A*	*D*—H	H···*A*	*D*···*A*	*D*—H···*A*
N1—H1A···O2^i^	0.89	2.04	2.909 (4)	166
N1—H1B···O1^ii^	0.89	2.03	2.875 (4)	159
N1—H1C···O3	0.89	2.02	2.857 (4)	156

Symmetry codes: (i) −*x*+2, −*y*, −*z*+1; (ii) −*x*+2, −*y*+1, −*z*+1.
